# The Ectopic Expression of Meiosis Regulatory Genes in Cutaneous T-Cell Lymphomas (CTCL)

**DOI:** 10.3389/fonc.2019.00429

**Published:** 2019-05-31

**Authors:** Jennifer Gantchev, Amelia Martínez Villarreal, Pingxing Xie, Philippe Lefrançois, Scott Gunn, Elena Netchiporouk, Denis Sasseville, Ivan V. Litvinov

**Affiliations:** Division of Dermatology, McGill University, Montréal, QC, Canada

**Keywords:** mycosis fungoides (MF), Sézary Syndrome (SS), cutaneous T-cell lymphomas (CTCL), cancer/testis antigens, germ cell proteins, meiCT, meiomitosis

## Abstract

Cancer testis (CT) antigens, under normal circumstances are uniquely expressed in testicular germ cells. Recent research has shown that meiosis-specific CT (meiCT) antigens are ectopically expressed in cutaneous T-cell lymphoma (CTCL) and may contribute to increased genomic instability. The aberrant activation of meiosis genes in a mitotic cell is now recognized as a distinctive process, “meiomitosis.” We have previously demonstrated the ectopic expression of several meiCT antigens in nine patient-derived CTCL cell lines and in expanded peripheral T lymphocytes isolated from Sézary Syndrome patients. In this study we analyzed the transcriptional expression of meiCT genes in Sézary Syndrome patients and healthy controls using publicly-available RNA sequencing (RNA-Seq) data. We corroborated our *in silico* analysis by examining the expression of 5 meiCT proteins in formalin-fixed, paraffin-embedded (FFPE) lesional samples from CTCL patients. Our results show significant differential gene expression of *STAG3, SGO2, SYCP3*, and *DMC1* in a cohort of Sézary Syndrome patients when compared to healthy controls. Additionally, our study demonstrates a heterogenous expression of meiCT genes involved in initiation (*STRA8*), sister chromatin cohesion (*STAG3, SGO2*), homologous chromosome synapsis (*SYCP3*) and homologous recombination (*DMC1*) in atypical lymphocytes in FFPE samples. Our results further confirm the ectopic expression of meiCT genes in CTCL which indicates that CTCL malignant cells likely undergo the process of cancer meiomitosis, as opposed to a typical mitotic division. The ectopic expression of meiCT genes together with investigations into the functional mechanisms of cancer meiomitosis will help provide a foundation to develop novel diagnostic tests to distinguish CTCL from benign inflammatory dermatoses and may enable us to develop additional targeted therapies for patients with this malignancy.

## Introduction

Cutaneous T-cell lymphoma (CTCL) is an extranodal non-Hodgkin's lymphoproliferative disease characterized by the infiltration of malignant T-cells into the skin (1). The two most commonly recognized forms of CTCL are mycosis fungoides (MF) and a leukemic variant, Sézary Syndrome (SS). MF, the predominant subtype of cutaneous lymphomas, presents with localized erythematous scaly patches and plaques on the trunk. Overall, this CTCL subtype has an indolent course and a good prognosis. MF patients often live >10–15 years with no further progression until the later stages of the disease. Histologically, the epidermis may show hyperkeratosis, acanthosis, the presence of Pautrier's microabscesses and a notable lack of significant spongiosis. Additionally, a dermal lichenoid infiltrate of atypical T lymphocytes (with atypical/cerebriform nuclei) and minimal papillary dermal fibrosis are often observed (2–4). SS is an aggressive subtype of CTCL with a poor prognosis and a mean disease-specific survival of only 2–4 years (5). SS patients present with erythroderma, pruritus, generalized lymphadenopathy, and circulating malignant T cells (6). The disease develops either *de novo* or evolves from an idiopathic erythroderma (7).

Genomic CTCL studies have been steadily increasing in number and revealed extensive chromosomal instability in malignant T cells from patients and patient-derived cell lines (8–10). In fact, an essential criterion for SS diagnosis is the presence of a chromosomally abnormal malignant T cell clone (11). Consistent with prior studies, we have characterized distinct genomic and transcriptional heterogeneity in MF and SS, that were demonstrated by widespread genomic alterations including translocations, insertions, deletions, and extensive copy number variations (9, 10, 12–16).

Current research has demonstrated the ectopic expression of a variety of CT antigens in CTCL, including the *MAGE* (17, 18), *CAGE* (19), and *GAGE* proteins (20) that confer several hallmarks of cancer including sustained growth, angiogenesis, evasion of apoptosis, tissue invasion, and metastasis (21). With regards to genomic instability, the family of CT antigens involved in meiosis is of particular interest. Genes that regulate meiosis are exclusively expressed during oocyte development/spermatogenesis and become transcriptionally silent in normal somatic tissues. Our lab has shown that several meiCT genes including *SPO11, REC8, SYCP3*, and *SYCP1* are ectopically expressed in CTCL (10, 22). We propose that the malignant lymphocytes in this cancer are undergoing meiomitosis, a process defined by the clashing of the mitotic and meiotic molecular machineries in malignancy. It has been proposed that the partial re-expression of meiotic genes could cause the increased genomic instability seen in dividing cancer cells (10, 23), but studies demonstrating this statement in human cancers are lacking.

The mechanisms driving meiotic gene expression in CTCL are largely unknown. The ectopic expression of meiotic proteins involved in crossing over, meiotic DSB formation and repair, may promote genomic rearrangements (21, 24), Moreover, mitotic expression of meiotic cohesins could promote aneuploidy by eliciting an aberrantly assembled mitotic spindle that can result in the shearing and mis-segregation of DNA (23, 25, 26). We have recently demonstrated the ectopic expression and function of meiotic genes in patient-derived CTCL cell lines representing both MF and SS, and in lymphocytes isolated from stage IV SS patients (10). We demonstrated that the ectopic expression of *STRA8, SYCP1, 3*, and other proteins in CTCL is tightly regulated throughout the cell cycle and that their expression is the result of a loss of epigenetic transcriptional repression. Moreover, we proposed that the extensive chromosomal instability seen in CTCL may result from the aforementioned ectopic expression of these normally restricted meiotic CT antigens (10). The current study aims to broaden the understanding of ectopic expression patterns of meiCT genes in CTCL by analyzing RNA-Seq expression data from Sézary Syndrome patients and corroborating these results in 32 histologically-confirmed CTCL skin samples.

## Methods

### Patients and Tissues

All patients were enrolled in this study with written informed consent and in accordance with the Declaration of Helsinki from The Ottawa Hospital (REB study #20150896-01H), McGill University Health Centre and affiliated hospitals (REB study #A09-M81-10A) and Laval University (REB study # 2011HES-22808). For immunohistochemistry, we analyzed a total of 32 samples from 29 patients with CTCL.

Diagnosis was histologically verified for all patients by a dermatopathologist and patients were staged according to the most recent consensus of the WHO-EORTC/ISCL staging system (5). Tissue samples were obtained by punch biopsy of the skin and were immediately frozen in liquid nitrogen, as previously described (22, 27, 28).

### Histology

Human skin biopsies were processed into formalin-fixed, paraffin-embedded (FFPE) tissue blocks using standard fixation and embedding techniques. All tissue samples were sectioned and prepared on positively charged slides. Immunohistochemistry staining was performed on FFPE tissue sections using the Leica Bond™ system and the standard protocol F. Sectioned slides were stained with the following anti-human antibodies: rabbit anti-*STRA8* (Novus Biologicals), rabbit anti-*STAG3* (Proteintech), rabbit anti-*DMC1* (Proteintech), rabbit anti-*SGO2* (Novus Biologicals), and rabbit anti-*SYCP3* (Abcam) using either heat mediated antigen retrieval with sodium citrate buffer (pH 6, epitope retrieval solution 1 ER1) for 20 min or with an EDTA buffer (pH 9, epitope retrieval solution 2 ER2) for 20 min. The sections were then incubated for 30 min at room temperature using the dilutions listed in Table S1 and detected using an HRP conjugated compact polymer system. Slides were then stained using DAB as the chromogen, counterstained with Hematoxylin, mounted, and cover slipped.

Slides were analyzed by a team of four investigators on a multi-head microscope (Nikon Eclipse Ni-U) with a 20X objective lens using a consensus scoring method. The investigators were blind to diagnosis and clinical stage of the sample. Positive cells were verified (using a 40x lens) for enlarged/atypical nuclei or cerebriform nuclear contours, characteristic of malignant T lymphocytes. Nuclear and/or cytoplasmic staining of irregular cells with characteristic malignant T cell morphology was considered positive staining. The percentage of positive cells were assigned as Zero−0% (negative); I = <5%; II = 5–10% and III = >10%. The intensity of staining was scored as 1 (weak), 2 (moderate), and 3 (strong). The scores for percentage and intensity were multiplied for each sample to give a final expression score of 1–4 (weak-moderate staining with 10% or less positive cells) or 6–9 (strong staining with >10% positive cells).

### Sézary Syndrome Patient Gene Expression and Analysis

Gene expression data for Sézary Syndrome patients was obtained from publicly-available RNA-Seq data (12, 16). The data from Choi et al. (12) and Ungewickell et al. (16) were pooled together to increase the sample size for analysis. Datasets were converted from TPM (transcripts per million) to a normalized rank. A non-parametric resampling method (Bootstrapping) was used to compute *p*-values comparing the means of non-parametric rank fraction values between Sézary patients and normal controls, as previously described (29). *P*-values were corrected for multiple hypothesis testing using a Bonferroni correction.

### Statistical Analysis

Fisher's Exact test (FET) was used to analyze categorical outcomes in the histological analysis for gene expression scoring results between MF Stage I, StageII-III and aggressive forms of CTCL (i.e., SS, primary cutaneous CD8^+^ positive aggressive epidermotropic T- cell lymphoma, peripheral T-cell lymphoma, or primary cutaneous gamma-delta T-cell lymphoma). All values were calculated using RStudio version 1.1.456. All statistical significance tests were two-sided. *P*-values <0.05 were considered statistically significant.

## Results

### Expression of meiCT Genes in Sézary Syndrome Patients From External Cohorts

**Figure 1:**
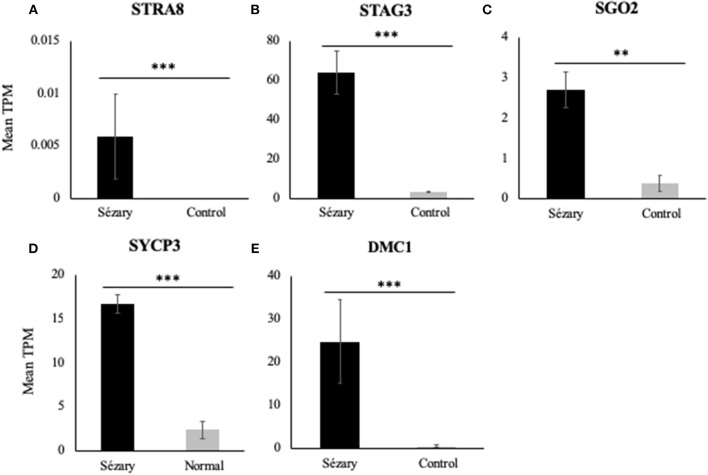
RNA-Seq gene expression of meiCT genes **(A)** *STRA8*, **(B)** *STAG3*, **(C)** *SGO2*, **(D)** *SYCP3*, and **(E)** *DMC1* in Sézary Syndrome patients compared to normal/control subjects based on pooled data from Choi et al. (12) and Ungewickell et al. (16) datasets. Data was normalized to a mean TPM value for each cohort. Asterisks indicate statistical significance.

Using RT-PCR, western blot analysis and immunofluorescence, we have previously shown that meiCT genes and proteins are ectopically expressed in nine patient-derived cell lines representing both MF and SS, and in lymphocytes isolated from three SS patients (10, 22). We wanted to expand those observations with a different method to attain patient data. We used publicly-available RNA-Seq datasets from two studies focusing on Sézary Syndrome patients (12, 16), as previously described (29). Mean transcripts per million (TPM) values were calculated for each gene for the SS and control groups for Choi et al. (12) and Ungewickell et al. (16) cohorts and then combined the values as one dataset for analysis. Mean TPM values were plotted on bar graphs to compare gene expression levels for Sézary Syndrome vs. normal control patients. Overall, we observed increased gene expression in Sézary patients for all tested genes (Figure 1). Using a rank-based non-parametric approach, these genes showed higher relative expression levels in SS patients compared to controls (Table 1; *p* < 10^−5^ for all genes except for *SGO2*; *p* = 0.035 for *SGO2*).

**Table 1:** RNA-sequencing results for differentially expressed meiCT genes in two pooled independent cohorts of Sézary Syndrome patients (12, 16).

**Genes**	**Ratio mean TPM SS/mean TPM Normal**	**Fraction Genes with TPM > GOI-Normal**	**Fraction Genes with TPM > GOI-Sézary**	***p*-value**
*STRA8*	∞	0	0.95	<10^−5^
*STAG3*	20.52	0.40	0.17	<10^−5^
*SGO2*	6.96	0.55	0.51	0.035
*SYCP3*	7.04	0.62	0.41	<10^−5^
*DMC1*	43.33	0.67	0.41	<10^−5^

*P-values were determined using non-parametric testing and are corrected for multiple hypothesis testing using Bonferroni's correlation. TPM, Transcripts Per Million; SS, Sézary Syndrome; GOI, Gene Of Interest*.

### Expression of meiCT Genes in CTCL Lesional Biopsies

To further support our RNA-Seq based statistical analysis of meiCT gene expression in SS patients, we used immunohistochemical staining of FFPE skin tissues biopsied from patients with different stages of CTCL, including SS. We analyzed IHC staining for 5 meiCT genes that are expressed at various stages of meiosis, ranging from initiation to homologous recombination, in 32 CTCL samples isolated form 29 patients. Patient age ranged from 21 to 90 years with a median of 65 ± 18 years with a male to female ratio of 1:1.9. Control samples included normal human skin, and human testis.

The percentage and intensity of positively stained cells of the atypical lymphoid infiltrate was recorded for each sample and for all five genes (Table 2). Positive cells displayed characteristic features of malignant T-lymphocytes including atypical/cerebriform nuclear contours and enlarged and/or hyperchromatic nuclei within the papillary dermis (mixed lichenoid infiltrate).

**Table 2:** Results of MeiCT gene expression in lesional skin biopsy samples of patients with CTCL.

**Gene**	**Disease**	**Expression score^**a**^ 1–4 <10% cells positive; weak-moderate staining**	**Expression score 6–9 ≥10% cells positive; moderate -strong staining**	***p*-value**
STRA8	MF Stage I	17/18	0/18	0.05
	MF Stage II/III	3/6	1/6	
	Aggressive^b^	5/7	1/7	
STAG3	MF Stage I	11/19	1/19	0.6
	MF Stage II/III	3/6	0/6	
	Aggressive^b^	2/7	0/7	
SGO2	MF Stage I	12/19	1/19	0.9
	MF Stage II/III	3/6	0/6	
	Aggressive^b^	5/7	0/7	
SYCP3	MF Stage I	16/19	0/19	1
	MF Stage II/III	5/6	0/6	
	Aggressive^b^	6/7	0/7	
DMC1	MF Stage I	9/19	10/19	0.2
	MF Stage II/III	4/6	2/6	
	Aggressive^b^	6/7	1/7	

*Expression score 1–4 corresponds to weak-moderate staining with <10% of positive cells. Expression score 6–9 corresponds to strong staining with ≥10% positive cells*.

a Intensity ^*^ Percentage = Expression score.

Intensity: 1 mild, 2 weak, 3 strong.

Percentage positive: 0, 0%; 1, ± and <5%; 2, 5–10%; 3 >10%.

b *Aggressive disease includes SS (n = 2), primary cutaneous gamma/delta T-cell lymphoma (n = 2), Primary cutaneous CD8^+^ positive aggressive epidermotropic T- cell lymphoma (n = 2) and Peripheral T-cell Lymphoma (n = 1)*.

**Figure 2:**
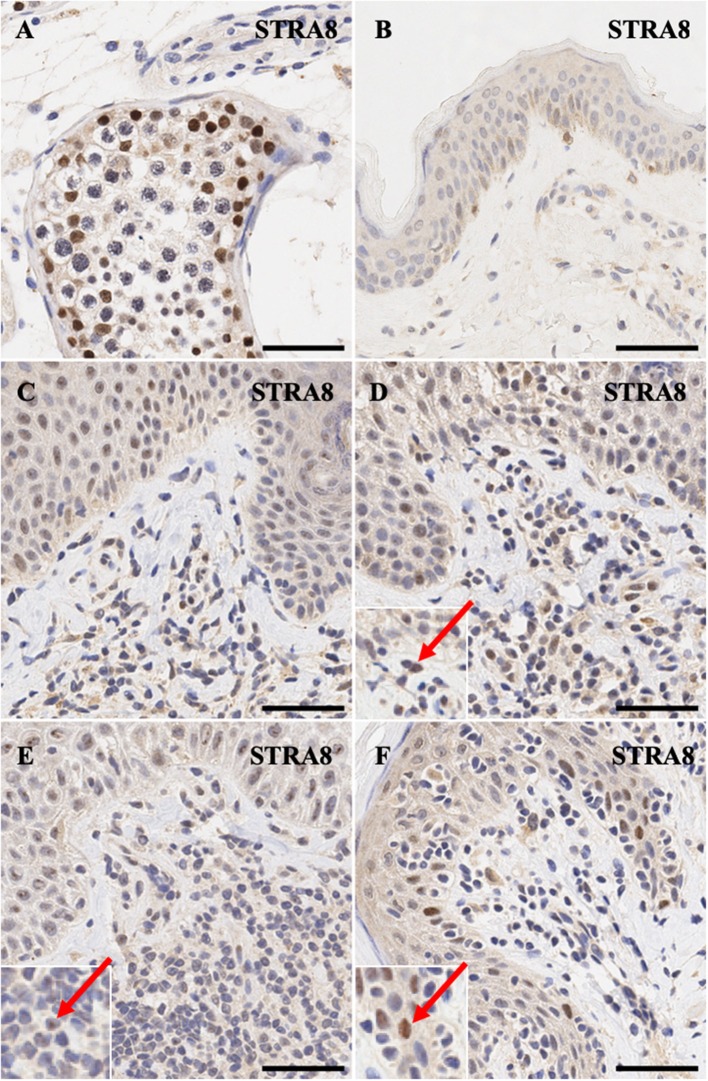
Immunohistochemistry staining of *STRA8* in **(A)** normal human testis (positive control), **(B)** normal skin **(C)** stage IB MF lesional skin, **(D)** stage IA MF lesional skin. **(E)** Sézary Syndrome **(F)** CD8^+^ MF lesional skin. Scale bars are 50 μm. Nuclear staining in malignant lymphocytes is highlighted (red arrow).

*STRA8* is an important of initiator of meiosis. Positive control expression of this gene in human testis shows intense nuclear expression pattern (Figure 2A). As expected, its expression is not detectable in normal skin except for diffuse non-specific background staining (Figure 2B). MF early stage samples and CD8^+^ MF (Figures 2C,D,F) show appreciable nuclear staining in a number of dermal atypical lymphocytes abutting the dermal-epidermal junction, while a number of atypical T cells in Sézary Syndrome patients exhibit specific nucleolar staining (Figure 2E).

**Figure 3:**
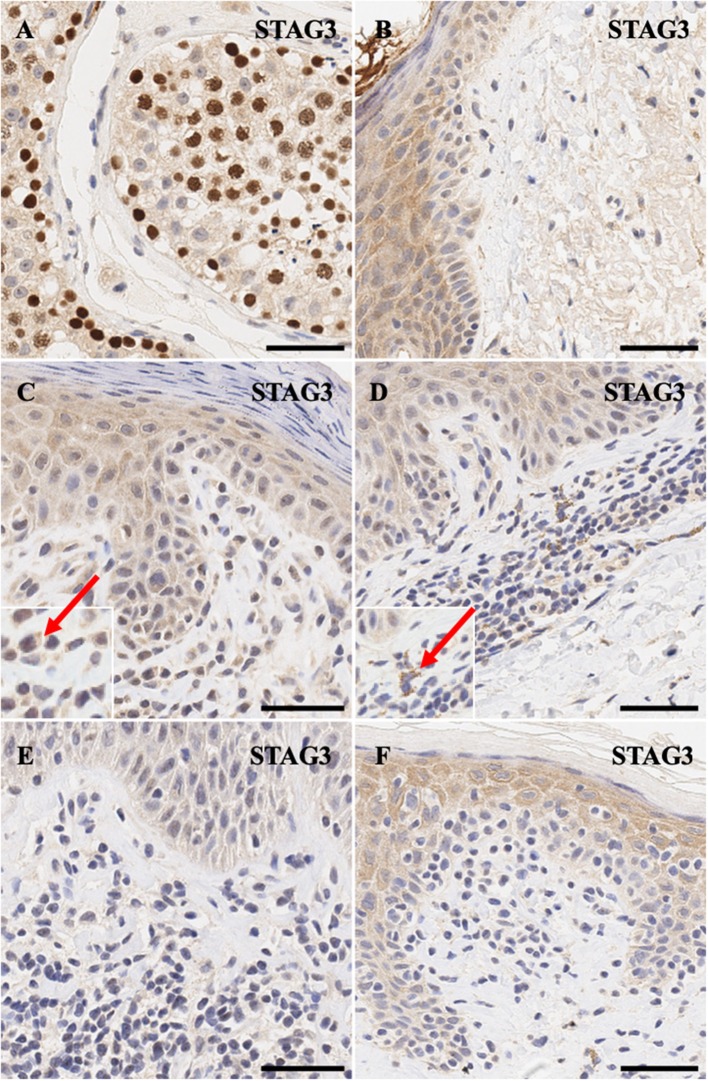
Immunohistochemistry staining of *STAG3* in **(A)** normal human testis (positive control), **(B)** normal skin **(C)** stage IIA MF lesional skin, **(D)** stage IB MF lesional skin. **(E)** Sézary Syndrome **(F)** CD8^+^ MF lesional skin. Scale bars are 50 μm. Red arrow highlights cytoplasmic staying of this protein in a number of cells.

**Figure 4:**
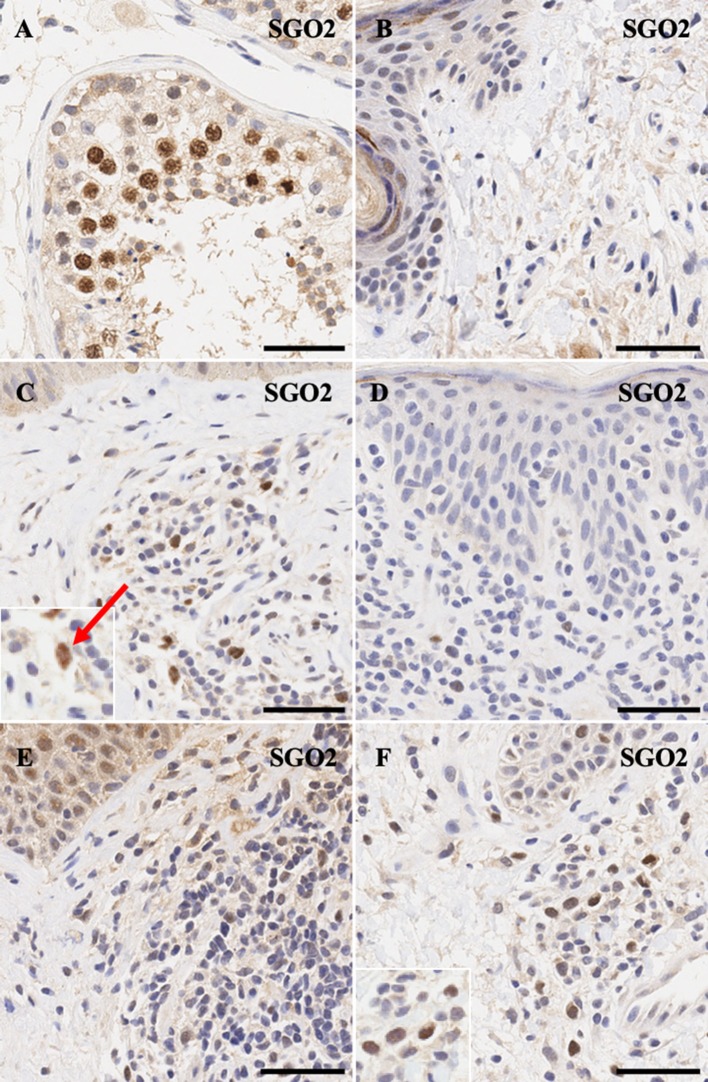
Immunohistochemistry staining of *SGO2* in **(A)** normal human testis (positive control), **(B)** normal skin **(C)** stage IIA MF lesional skin, **(D)** CD8^+^ MF lesional skin, **(E)** Sézary Syndrome (**F)** peripheral T-Cell Lymphoma. Scale bars are 50 μm. Nuclear staining in malignant lymphocytes is highlighted (red arrow).

Similarly, meiotic cohesion proteins *STAG3* (Figures 3A,B) and *SGO2* (Figures 4A,B) demonstrate mostly nuclear staining in the testis and background non-specific staining in the epidermal keratinocytes, respectively. More precisely, *STAG3* shows intense non-specific staining in the granular layer, while *SGO2* shows diffuse staining throughout the epidermis and intense staining in the corneal layer of normal skin. Overall, staining for *STAG3* was mostly weak and ranged from pan-cellular expression pattern (Figure 3C) to mostly cytoplasmic (Figure 3D). In some cases, only faint nucleolar staining was observed (Figures 3E,F). In contrast, *SGO2* staining was more robust and demonstrated specific nuclear expression in a number of atypical cells (Figures 4C–F).

**Figure 5:**
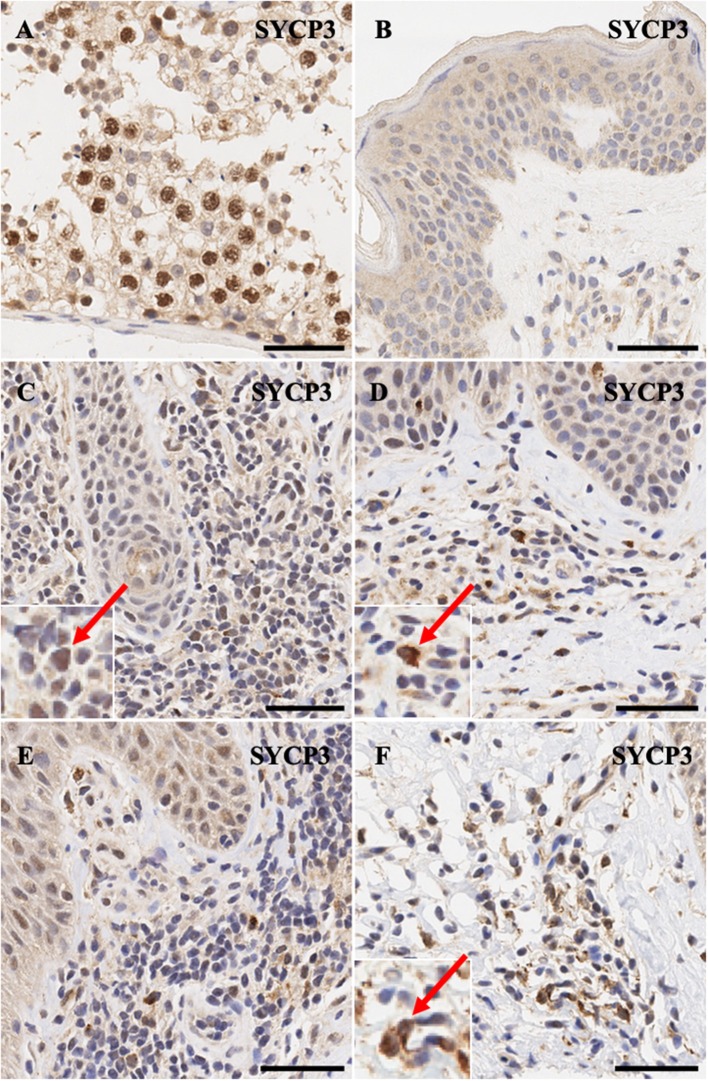
Immunohistochemistry staining of *SYCP3* in **(A)** normal human testis (positive control), **(B)** normal skin **(C)** stage IB MF lesional skin, **(D)** stage IB MF lesional skin, **(E)** Sézary Syndrome **(F)** stage IA MF lesional skin. Scale bars are 50 μm. Nuclear>cytoplasmic staining in malignant lymphocytes is highlighted (red arrow).

Synaptonemal complex protein *SYCP3* is expressed predominantly in the nuclei>cytoplasm of spermatogonia (Figure 5A) and only non-specific background staining is observed in normal skin (Figure 5B). Notably, atypical lymphocytes in CTCL express *SYCP3* in a nuclear and in some cases mis-localized pan-cellular pattern (Figures 5C–F).

**Figure 6:**
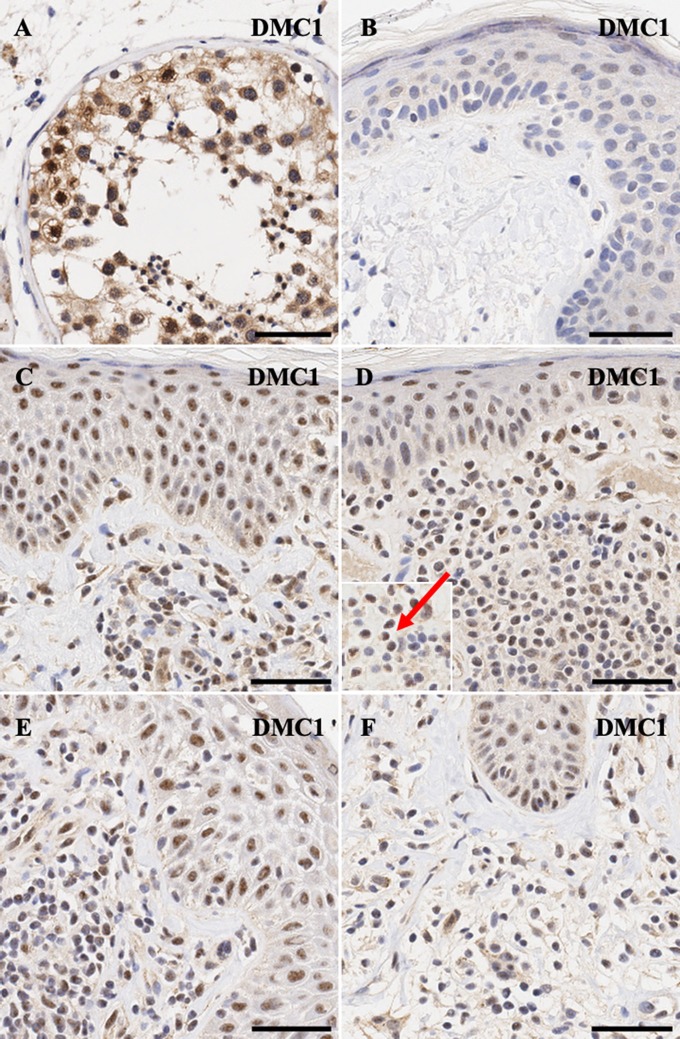
Immunohistochemistry staining of *DMC1* in **(A)** normal human testis (positive control), **(B)** normal skin **(C)** stage IA MF lesional skin, **(D)** stage IA MF lesional skin, **(E)** Sézary Syndrome **(F)** CD8^+^ MF lesional skin. Scale bars are 50 μm. Nuclear staining in malignant lymphocytes is highlighted (red arrow).

Finally, for *DMC1*, meiotic recombination protein, nucelar>cytoplasmic expression is seen in spermatogonia (Figure 6A), while malignant lymphocytes in CTCL demonstrate strong nuclear >>> cytoplasmic expression for this protein (Figures 6C–F).

Detailed scoring for all genes across all lesional CTCL skin samples is presented in Table 2. While most proteins appear to be upregulated only weakly across different stages, *DMC1* shows robust staining across samples from all clinical stages of CTCL. Unfortunately, due to limited sample size, we did not observe meaningful differences between protein expression and clinical staging of patients (Table 2).

## Discussion

There is a unique set of meiosis specific genes that are temporally activated in germ cell development and are subsequently silenced in somatic cells. In this study we expanded our previous research by performing statistical analysis on a combined RNA-Seq dataset from Choi et al. (12) and Ungewickell et al. (16) that confirmed the differential expression of *STRA8, STAG3, SGO2, SYCP3*, and *DMC1* genes in Sézary patients when compared to healthy controls. We further corroborated our analysis with protein expression patterns of the meiCT genes in stained FFPE lesional skin samples isolated from patients diagnosed with various stages and subtypes of CTCL. We have shown that most of these proteins are expressed in atypical lymphocytes, albeit at weak to moderate levels. However, this pattern is expected for tightly-regulated meiCT genes whose expression occurs at specific time points in actively dividing cells. Furthermore, *DMC1* showed robust strong expression in biopsy samples representing different stages of CTCL. Our findings corroborate and extend our recent work regarding the exploration of the ectopic expression and functional analysis of meiCT antigens in CTCL (10).

Under normal circumstances, *STRA8* is a vital protein to initiate meiosis in spermatocytes/oocytes in response to RA (retinoic acid) signaling (30–36). STRA8 expression is upregulated when germ cells switch from a mitotic state to meiosis (35) and spermatocytes that are depleted of STRA8 fail to enter meiosis. However, the role of STRA8 in cancer is yet to be defined. In germ cells, *STRA8* protein is primarily localized to the cytoplasm. In this study, we demonstrated that STRA8 is heterogeneously expressed in the nucleus of malignant T lymphocytes from CTCL patients. Other studies have shown expression of this protein in the nucleus with potential double stranded DNA binding (37, 38). Hence, STRA8 may have transcription regulatory activity (38) given reports that STRA8 expression is found in the nucleus of primordial germ cells and teratocarcinoma cell lines (37, 38). Additionally, two studies have shown that STRA8 binds double-stranded DNA (37, 38) and may have a role in transcription (38).

Throughout meiosis, a number of proteins form complexes along sister chromatids to orchestrate cohesion and release in a tightly regulated process to ensure the proper distribution of chromatids into gametes. The meiosis-specific cohesin subunit protein, *STAG3* (*Stromal antigen 3*), binds sister chromatids arms in meiosis I (39) and provides stability of meiosis-specific cohesion complexes (40). The cohesion complex is prudently protected by *SGO2* (*Shugoshin 2*) to avoid untimely degradation by separases. Although *SGO2* can also be expressed in mitotic cells, *SGO2* deficient mice form aneuploid gametes that result in infertility (41). However, lack of *SGO2* does not affect cohesion formation or development in cultured adult somatic cells and fibroblasts (41). We observed positive IHC staining of the two cohesion molecules in <5% of the atypical lymphoid infiltrate present in the papillary dermis. These results are not surprising since few T-cells are actively undergoing cell division at any given time in CTCL.

Similar results were found for the synaptonemal complex protein *SYCP3*. *SYCP3* forms the synaptonemal complex (SC) which is considered one of the cardinal features of meiosis. The SC a highly ordered assembly of proteins that comprise the interface between paired homologous chromosomes in meiosis. This meticulous complex stabilizes the interactions between homologs through a process called synapsis and promotes recombination (42). Staining of *SYCP3* was found in <5% of atypical lymphocytes in the papillary dermis.

As meiosis progresses, *DMC1* (Disrupted Meiotic cDNA 1) catalyzes strand exchange by localizing to double-stranded DNA (DSB) break sites and facilitates efficient homologous recombination DNA repair (43). *DMC1* localizes to meiotic DNA break sites to initiates strand exchange (43) and was expressed in 10–15% of the lymphoid infiltrate. We hypothesize that the expression of meiotic genes is tightly regulated and temporally expressed by the malignant T-cell in CTCL patients. Based on our previous work, we have demonstrated the temporal expression of meiCT genes at the onset of the cell cycle in patient-derived cell lines (10). Therefore, our IHC positive cells have the potential to undergo meiomitotic cell division.

The transcriptional activation of meiCT genes is not fully understood, however studies have demonstrated two mechanisms of reactivation: the hypomethylation of meiCT gene promoter regions in lung adenocarcinoma (44), and increased expression of select meiCT genes following a genotoxic stress in cervical cancer, melanoma and lymphoma cell lines (25, 45, 46). Studies have demonstrated the ectopic expression of meiCT genes in polyploid lymphoma and cervical cells during depolyploidization (25, 45). CTCL cells may express meiCT genes to promote survival by maintaining stable ploidy and the proliferative states of the malignant T cells. Additionally, the concurrent activation of both the meiotic and mitotic pathways may represent an adapted regulatory pathway for a reductional division.

The expression of the homologous recombination *DMC1*gene and cohesin *SGO2* factor have the propensity to contribute to genomic rearrangements and increase aneuploidy, another potential mechanism for survival in a dynamic lesional/tumor environment. Previous studies have shown that CT antigens have the potential to modulate chromosomal aberrations and contribute to increased genomic instability (10, 47). Deregulation or even the presence of the meiotic proteins like *DMC1* may initiate a partial meiotic program in mitotic cells and have the potential to influence genomic translocations, insertions and deletions during cell division.

We would like to address an important limitation, that due to a relatively small sample size of biopsy samples, we did not detect a correlation between CTCL clinical disease stage and protein expression from IHC staining. Future studies with a larger sample size would be able to estimate expression levels more precisely across different CTCL stages for each protein. In addition, another limitation is the semi-quantitative nature of IHC staining. Due to the variable staining patterns we adopted a semi-quantitative consensus scoring method. Unfortunately, we were not able to provide a quantitative analysis of the analyzed IHC stained slides.

In conclusion, the ectopic expression of meiCT genes in CTCL raises the possibility that malignant infiltrating T lymphocytes are undergoing a process termed meiomitosis. Further studies of the functional mechanisms of meiCT genes might shed light on the role of meiomitosis in carcinogenesis for various malignancies including CTCL. Further investigation of meiCT protein roles in carcinogenesis may lead to the development of small molecule inhibitors targeting this aberrant meiomitosis cell division. This can ultimately lead to the development of biomarkers for CTCL and novel targeted therapies.

## Ethics Statement

All patients were enrolled in this study with written informed consent and in accordance with the Declaration of Helsinki from The Ottawa Hospital (REB study #20150896-01H), McGill University Health Centre and affiliated hospitals (REB study #A09-M81-10A) and Laval University (REB study # 2011HES-22808).

## Author Contributions

JG, AM, SG, and PL performed histological scoring. PL and PX performed the data acquisition and analysis of Sézary patient cohort. PL, PX, and JG performed statistical analyses. AM and JG prepared the figures. JG wrote the first draft of the manuscript. All authors contributed to the revision and approval of the submitted manuscript. IV, EN, and DS contributed to the conception and design of the study.

### Conflict of Interest Statement

The authors declare that the research was conducted in the absence of any commercial or financial relationships that could be construed as a potential conflict of interest.
