# Supplementary material for: The Ectopic Expression of Meiosis Regulatory Genes in Cutaneous T-Cell Lymphomas (CTCL)
Source: Front Oncol. 2019 May 31;9:429. doi: 10.3389/fonc.2019.00429 (PMC6554469; doi:10.3389/fonc.2019.00429)
Supplement: Supplementary file 1 [file Table_1.DOCX]

Table S1. Immunohistochemistry (IHC) antibodies and dilutions.

| **Antibody Name** | **Antigen Retrieval Method** | **Dilution Used** | **Positive Control(s)** | **Negative Control** |
| --- | --- | --- | --- | --- |
| **Rabbit DMC1**  Proteintech #13714-1-AP | ER1 20 min | 1:200 | Human Testis, H9 Tumor | Human Skin |
| **Rabbit SGO2**  Novus #NBP1-83567 | ER1 20 min | 1:100 | Human Testis, H9 Tumor | Human Skin |
| **Rabbit STRA8**  Abcam #ab49602 | ER 1 20 min | 1:500 | Human Testis, H9, and PB2B Tumor | Human Skin |
| **Rabbit STAG3**  Proteintech #23314-1-AP | ER1 20 min | 1:200 | Human Testis, H9, and PB2B Tumor | Human Skin |
| **Rabbit SYCP3**  Abcam #ab15093 | ER1 20 min | 1:400 | Human Testis, H9, PB2B Tumor | Human Skin |
